# Commentary: Grading of clear cell renal cell carcinoma using diffusion MRI with a multimodal apparent diffusion model

**DOI:** 10.3389/fonc.2026.1754044

**Published:** 2026-01-26

**Authors:** Jing Chen, Pengpeng Huang, LiFang Yang

**Affiliations:** 1 Center for Rehabilitation Medicine, Rehabilitation & Sports Medicine Research Institute of Zhejiang Province, Department of Rehabilitation Medicine, Zhejiang Provincial People’s Hospital (Affiliated People’s Hospital), Hangzhou Medical College, Hangzhou, Zhejiang, China; 2 Urology & Nephrology Center, Department of Urology, Zhejiang Provincial People’s Hospital (Affiliated People’s Hospital), Hangzhou Medical College, Hangzhou, Zhejiang, China

**Keywords:** clear cell renal cell carcinoma, imaging biomarker standardization, preoperative risk stratification, standardization and reproducibility, tumor grading

We read with great interest the article by Wang et al. proposing a multimodal apparent diffusion (MAD) model based on diffusion-weighted MRI (DWI) for preoperative grading of clear cell renal cell carcinoma (ccRCC) (1). This work is particularly timely given the rapid expansion of advanced MRI biomarkers in urological oncology, where noninvasive and biologically interpretable tools are increasingly expected to refine preoperative risk stratification and guide individualized management. ccRCC is the dominant renal malignancy subtype, and WHO/ISUP grade is strongly associated with prognosis, recurrence, and treatment intensity; nevertheless, dependable preoperative grading remains an unmet clinical need (2, 8).

In a retrospective cohort of 54 ccRCC cases scanned using a broad b-value protocol (0–3000 s/mm²), the authors decomposed the DWI signal into restricted, hindered, unimpeded diffusion, and flow-related components. High-grade tumors (WHO/ISUP 3–4) demonstrated lower hindered diffusion coefficient (Dh) and higher restricted fraction (fr) and heterogeneity parameter (ah), while ADC also decreased; a combined Dh+fr+ah model achieved an AUC of 0.796 and outperformed ADC (1). Such multi-compartment and non-Gaussian diffusion strategies are consistent with emerging renal oncology imaging trends aimed at capturing microstructural complexity beyond mono-exponential ADC (3–5).

Several methodological and translational issues deserve attention before MAD-DWI can mature into a clinically robust diagnostic strategy. The most significant confounding factor is the imbalance of T-stage between groups: low-grade tumors were predominantly T1, whereas high-grade lesions tended to present with T3/T4 disease. Advanced diffusion parameters (such as Dh, fr, and ah) are known to correlate not only with grade but also with features that evolve with stage progression, including increasing tumor size, necrosis, and microvascular or stromal remodeling (3, 4, 6). Consequently, without stage-matched validation, the currently observed differences may reflect a composite signature of both tumor stage and grade, making it difficult to disentangle a true “grade signature.” Clarifying this distinction is critical for translation because T-stage is already reliably assessable on routine contrast-enhanced CT or MRI, whereas accurate preoperative grading remains the key unmet need. Future studies should prioritize stage-adjusted multivariable modeling or stratified analyses within comparable subsets—especially T1 tumors where nephron-sparing decisions are sensitive to grading—to strengthen the specificity of MAD metrics (3, 6).

Building on the reviewer’s suggestion, we have expanded our commentary to recommend that future MAD-DWI studies include stage-adjusted multivariable analyses and stage-matched subgroup comparisons, particularly within T1 lesions where treatment decisions (e.g., active surveillance vs. nephron-sparing surgery) are most sensitive to accurate grading. Such methodological refinements would more precisely define whether MAD-derived metrics truly capture grade-specific microstructural features independent of tumor stage.

ROI representativeness constitutes another bottleneck. The study relied on a single largest solid-slice ROI while excluding necrosis, hemorrhage, and calcification. Although this increases fitting stability, ccRCC is biologically and spatially heterogeneous, and grade often varies across tumor regions. Single-slice sampling risks under-representing whole-lesion risk and may bias estimates toward more uniform viable tissue, particularly in high-grade tumors where necrosis and mixed architecture are common. Multi-regional and whole-tumor radiomics studies consistently show that capturing intratumoral and peritumoral heterogeneity improves correlation with WHO/ISUP grade and prognosis, reinforcing that global lesion characterization is essential for emerging diagnostics (7–9). In this context, the observed increase of ah with grade—interpreted as greater structural uniformity in high-grade lesions—may reflect ROI exclusion of heterogeneous necrotic fractions rather than a genuine biological inversion. Three-dimensional segmentation or multi-slice ROIs, followed by voxel-wise histograms or heterogeneity indices, would likely clarify this relationship and better align MAD readouts with the clinical question of entire-tumor aggressiveness (7–9).

Standardization and reproducibility remain decisive for multi-center adoption. MAD fitting depends on broad b-value sampling and multi-parameter optimization, and advanced diffusion models are sensitive to scanner hardware, sequence timing, b-value distributions, motion correction, and fitting constraints (3–6, 10). ADC in the current study was fitted across the full 0–3000 s/mm² range, differing from typical renal DWI practice and limiting comparability with established ccRCC grading literature (1, 5, 6). We encourage fuller reporting of fitting uncertainty (e.g., confidence intervals, residuals), sensitivity testing using alternative b-value subsets, and inter-/intra-reader ROI reproducibility, alongside harmonization efforts across platforms. Such steps are prerequisites if MAD parameters are to serve as stable thresholds within real-world urological oncology workflows.

To further clarify the potential clinical value of MAD-DWI, we now explicitly distinguish between the respective roles of staging and grading in the preoperative assessment of renal masses. Tumor stage, including local extension beyond the kidney and involvement of perinephric structures, is readily identifiable on standard cross-sectional imaging and already guides major treatment pathways. In contrast, reliable noninvasive grading remains elusive, despite its central importance for prognostication, selection of nephron-sparing approaches, and determining eligibility for active surveillance.

Therefore, the added value of MAD-based diffusion modeling lies not in improving staging—which existing imaging already accomplishes—but rather in its potential to provide a biologically interpretable, noninvasive grading biomarker. Within real-world workflows, MAD-DWI could complement routine imaging by refining risk stratification, assisting decision-making when biopsy is inconclusive or not feasible, and offering microstructural insight that may improve preoperative planning.

These clarifications strengthen the conceptual framing of MAD as a grading-focused adjunct rather than a staging tool.

Overall, Wang et al. present an important contribution toward biologically grounded, noninvasive ccRCC grading in line with this specialty section’s aims. Strengthening grade specificity independent of stage, extending analysis to whole-tumor heterogeneity, and prioritizing standardization will help MAD-DWI evolve from a promising model into a truly actionable emerging diagnostic strategy for renal cancer.
